# Multimodality imaging in hypertrophic cardiomyopathy

**DOI:** 10.1186/s44348-025-00060-7

**Published:** 2026-01-07

**Authors:** Jihoon Kim, Sang-Chol Lee

**Affiliations:** Division of Cardiology, Department of Internal Medicine, Heart Stroke Vascular Institute, Samsung Medical Center, Sungkyunkwan University School of Medicine, Seoul, Republic of Korea

**Keywords:** Hypertrophic cardiomyopathy, Imaging, Magnetic resonance imaging, Computed tomography

## Abstract

Hypertrophic cardiomyopathy (HCM) is a myocardial disorder characterized by unexplained myocardial hypertrophy. Although the diagnosis of HCM is traditionally based on increased left ventricular (LV) wall thickness, contemporary management requires a comprehensive multimodality imaging approach to accurately define disease phenotype, assess functional consequences, and guide risk stratification. Transthoracic echocardiography remains the first-line imaging modality, providing real-time evaluation of LV morphology, systolic and diastolic function, and LV outflow tract obstruction (LVOTO). However, its ability to assess myocardial tissue characteristics and complex morphologic variants may be limited in selected patients. Cardiac magnetic resonance (CMR) offers superior spatial resolution and allows detection of myocardial fibrosis using late gadolinium enhancement. Cardiac computed tomography serves as a complementary tool for evaluating coronary artery anatomy and detailed cardiac structure, particularly in patients with suboptimal echocardiographic windows or contraindications to CMR. This review summarizes the strengths and limitations of each imaging modality and highlights their complementary roles in the evaluation of cardiac morphology, systolic and diastolic function, LVOTO, and tissue characterization. An integrated imaging strategy is essential for optimized diagnosis, individualized risk stratification, and informed therapeutic decision-making in patients with HCM.

**Supplementary Information:**

The online version contains supplementary material available at 10.1186/s44348-025-00060-7.

## Background

Hypertrophic cardiomyopathy (HCM) is a relatively common inherited disorder characterized by thickening of the myocardium in the absence of abnormal loading conditions. Although the diagnosis of HCM is primarily based on increased left ventricular (LV) wall thickness, the disease presents with diverse morphological phenotypes and hemodynamic profiles [1]. Therefore, appropriate imaging modalities play a central role in the comprehensive evaluation of both suspected and confirmed HCM cases. Transthoracic echocardiography (TTE) is typically the first-line tool, as it provides immediate assessment of structural and functional parameters, including myocardial thickness, systolic and diastolic function, and LV outflow tract obstruction (LVOTO). Additionally, cardiac magnetic resonance (CMR) offers precise measurements of cardiac size and function, while also detecting myocardial fibrosis or scarring, which may influence clinical outcomes. Cardiac computed tomography (CT) can be useful for evaluating coronary artery anatomy or clarifying structural abnormalities not well visualized on echocardiography or CMR. Each imaging modality should be selected based on the specific clinical question and the inherent strengths of the technique.

## Cardiac morphology

### Diagnosis

The diagnosis of HCM can be established by a maximal end-diastolic wall thickness of ≥ 15 mm in any segment of the LV myocardium, provided that the hypertrophy is not explained by other conditions [2, 3]. Therefore, accurate measurement of LV wall thickness and careful differentiation from other causes of myocardial thickening are essential first steps in the imaging evaluation of patients with suspected HCM.

**Fig. 1:**
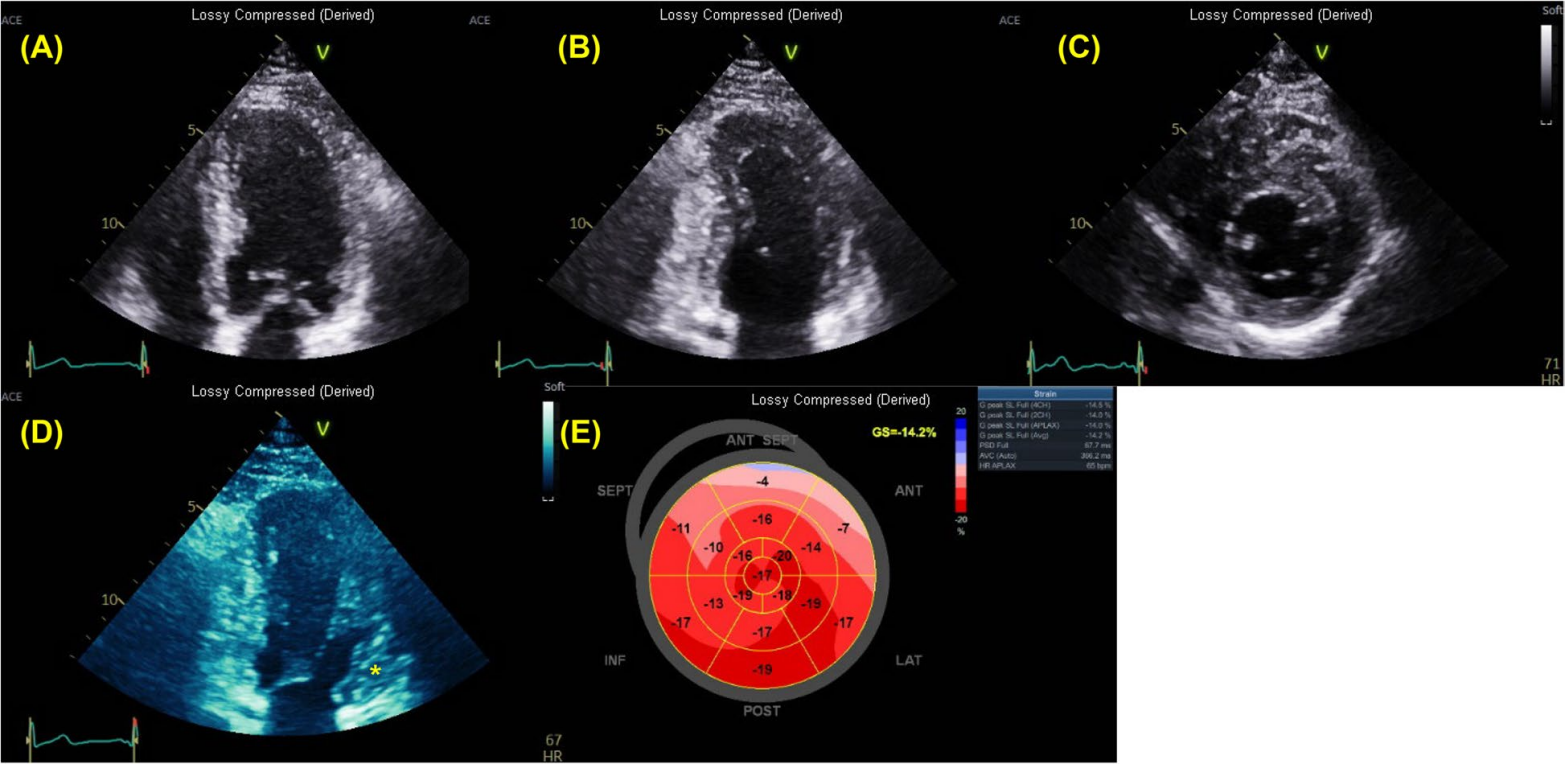
Transthoracic echocardiography of 38-year-old male patient with hypertrophic cardiomyopathy. No definite myocardial hypertrophy is observed in the (**A**) apical four-chamber and **B** two-chamber views. **C** However, thickening of the basal anterior wall is noted in the parasternal short-axis view. **D** A slightly adjusted apical two-chamber view demonstrates basal anterior wall hypertrophy (asterisk). **E** Longitudinal strain is reduced in the basal anteroseptal and anterior walls

**Fig. 2:**
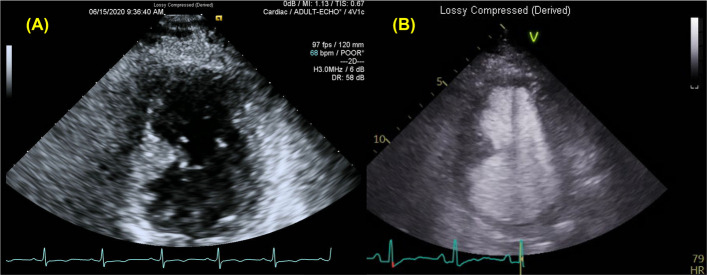
Contrast echocardiography. **A** Transthoracic echocardiography revealing thickening of the mid-inferoseptal wall, while the apical myocardium not being clearly visualized. **B** Following contrast injection, the apical myocardium becomes well visualized

TTE is the first-line imaging modality for evaluating cardiac morphology in patients with suspected HCM due to its accessibility, real-time imaging capability, and ability to provide detailed structural and functional information. Accurate measurement of LV wall thickness should be performed in multiple views, including parasternal long- and short-axis and apical window, to avoid missing localized or atypical patterns of hypertrophy (Fig. 1). The septum is the most frequently involved region, and asymmetrical septal hypertrophy remains the most common phenotype [4]. Nonetheless, echocardiographers must remain vigilant for nonclassical patterns such as apical, mid-ventricular, or lateral wall hypertrophy, which may require off-axis imaging for better visualization. Meticulous care is also essential to avoid overestimation, particularly in areas with oblique imaging planes or poor endocardial definition, or in cases of sigmoid septum, which is frequently observed in elderly. In some cases, echocardiographic contrast agents can enhance endocardial border delineation, especially in patients with suboptimal image quality or suspected apical involvement (Fig. 2) [5].

**Fig. 3:**
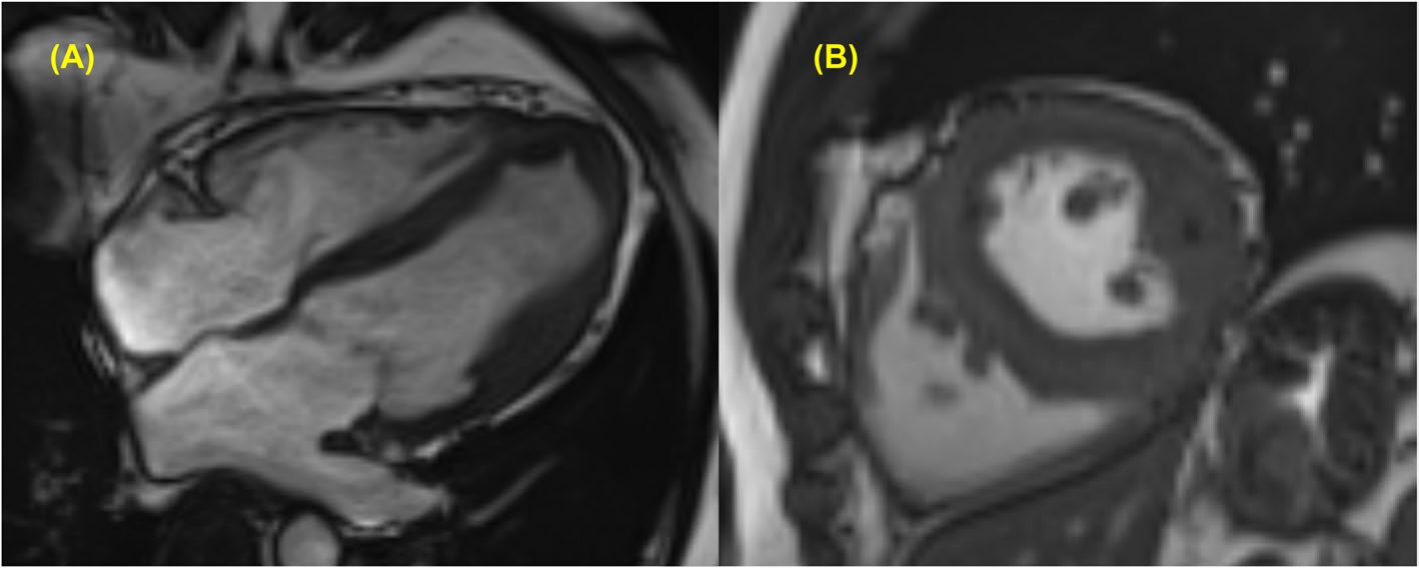
Cardiac magnetic resonance images showing atypical presentations of hypertrophic cardiomyopathy. Hypertrophied myocardium of the mid-anterolateral wall is observed in both (**A**) the apical four-chamber view and **B** the parasternal short-axis view

Although TTE has long been the gold standard for the clinical diagnosis of HCM, CMR imaging offers superior spatial resolution and high-quality tomographic images for detailed assessment of cardiac structure and morphology. CMR is known to detect both the presence and the maximal extent of LV hypertrophy more frequently than echocardiography [6]. It is particularly useful for identifying segmental or atypical patterns of hypertrophy, such as anterolateral wall thickening, which may be underdiagnosed on TTE (Fig. 3) [6]. Current clinical guidelines recommend the use of CMR in patients with poor echocardiographic image quality or inconclusive findings [2]. In addition to wall thickness measurement, CMR enables detailed visualization of structural abnormalities such as abnormal papillary muscles and mitral valve apparatus, right ventricular involvement, and provides accurate quantification of myocardial mass. However, caution is needed to avoid overestimation of LV wall thickness, especially when measured in long-axis view where foreshortening may occur. The feasibility of CMR can be limited by several factors, including the presence of implanted cardiac devices, high cost, and limited availability in some clinical settings.

### Apical hypertrophy or aneurysm

**Fig. 4:**
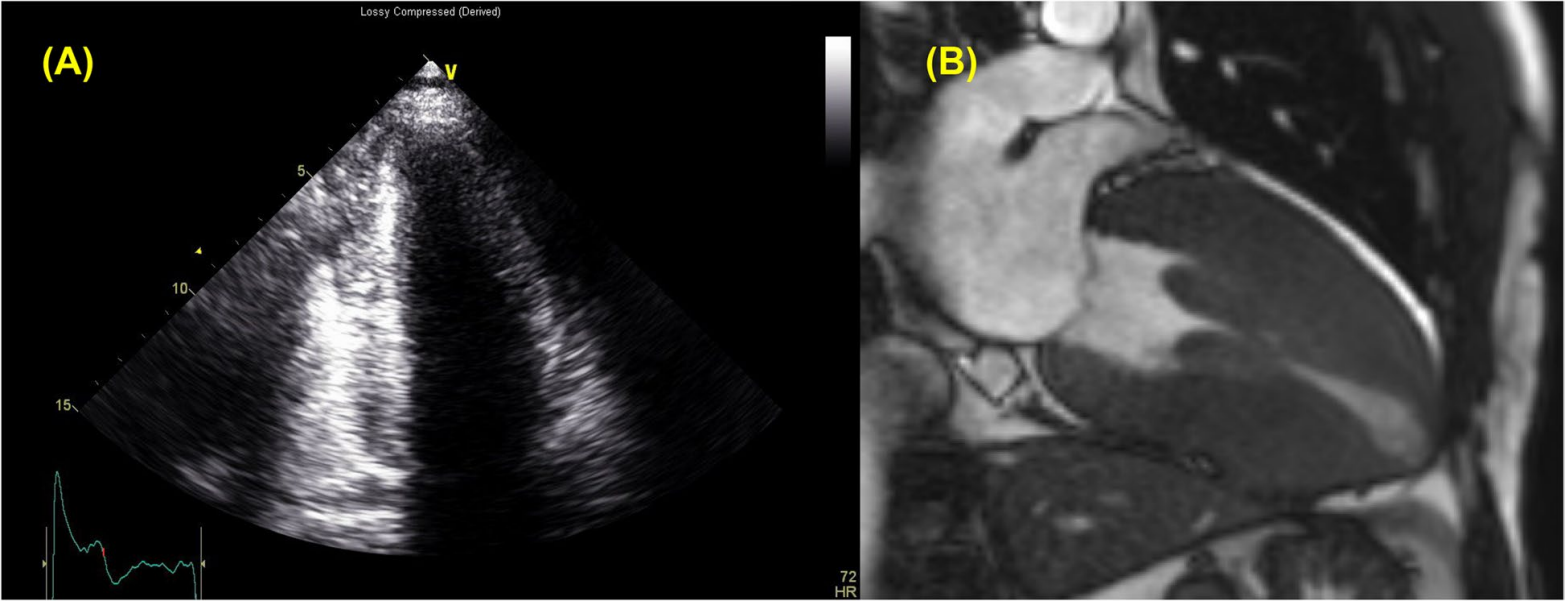
Patients with apical aneurysm. Compared to the suboptimal image quality of (**A**) transthoracic echocardiography, **B** cardiac magnetic resonance imaging clearly reveals a small apical aneurysm during the systolic phase

In HCM, hypertrophy may be confined to the LV apex (pure form) or may be most prominent at the apex (mixed form) [7]. The prevalence of this phenotype, apical HCM, varies widely across ethnic groups. In Western populations, it has been reported in 1% to 10% of HCM cases, whereas in Asian populations, the prevalence may be as high as 25% [8, 9]. Some patients exhibited LV cavitary obliteration, with or without apical aneurysm, the latter being a known risk factor for adverse clinical outcomes. CMR enables more reliable detection of apical hypertrophy and aneurysms, which may be missed on TTE (Fig. 4) [10–12].

### Cardiac CT

Cardiac CT provides high-resolution imaging of cardiac morphology and can serve as a useful complementary tool. It is particularly useful for noninvasive coronary artery assessment. Additionally, cardiac CT enables detailed three-dimensional reconstruction of cardiac anatomy and precise localization of septal branches of the coronary arteries, which can assist in procedural planning for septal reduction therapy.

## Assessment of systolic function

### Ejection fraction

LV systolic function is a significant prognostic factor in patients with HCM. Echocardiography remains a cornerstone for evaluating LV systolic function. In most patients with HCM, the LV ejection fraction (LVEF) is preserved; however, a reduced LVEF < 50% is observed in approximately 4% to 8% of cases and is strongly associated with HCM-related adverse events as well as all-cause mortality [13, 14]. Furthermore, even a low normal LVEF (50%–55%) has been identified as an independent predictor of heart failure and cardiovascular death [15]. Therefore, accurate measurement of LVEF is essential for appropriate risk stratification in patients with HCM. Tissue Doppler myocardial imaging, including mitral annular systolic velocity (s’), may also provide additional prognostic information, but its utility can be limited by the heterogenous distribution of myocardial hypertrophy [16].

### Myocardial strain

**Fig. 5:**
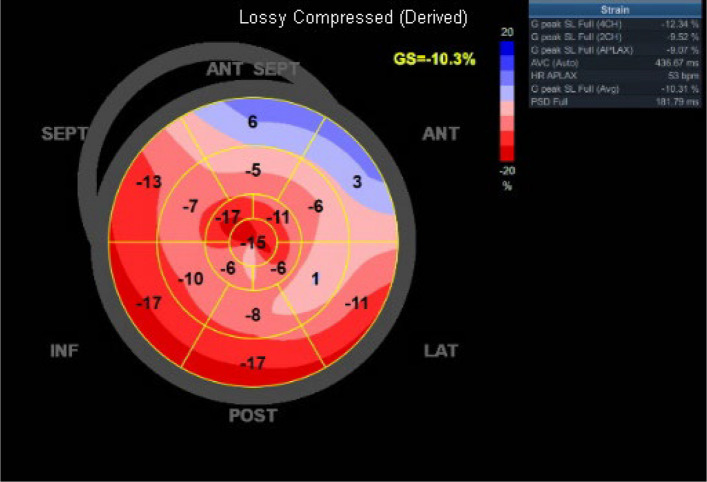
In this patient with hypertrophic cardiomyopathy, left ventricular ejection fraction was 61.4%; however, longitudinal strain was reduced to –10.3%

Two-dimensional speckle tracking echocardiography can detect subclinical LV systolic dysfunction and provides additional prognostic value in patients with HCM. LV longitudinal strain can be reduced even in patients with preserved LVEF, reflecting early myocardial impairment (Fig. 5, Video 1). Among strain parameters, global longitudinal strain is particularly well established and has been known to predict adverse outcomes beyond traditional risk factors in HCM [17–19]. Incorporating LV longitudinal strain into current risk stratification models may improve the prediction of sudden cardiac death [20].

CMR is a valuable tool for evaluating LV systolic function, particularly in patients with poor echocardiographic image qualities. Similar to speckle tracking echocardiography, a feature-tracking technique has been introduced in CMR to assess myocardial strain, which may assist in detecting subclinical dysfunction and differentiating HCM from other causes of myocardial hypertrophy [21, 22].

## Assessment of diastolic function

### Predicting LV filling pressure

The LV hypertrophy, myocardial fibrosis, and ischemia observed in HCM contribute to the development of LV diastolic dysfunction (LVDD), a hallmark feature of HCM, which often contributes to heart failure symptoms. Although echocardiography is widely used to assess LVDD, its diagnostic accuracy has known limitations in patients with HCM. In a study comparing echocardiographic parameters with invasive hemodynamic measurements, the E/e′ ratio showed only a modest correlation with mean left atrial (LA) pressure measured by cardiac catheterization [23]. Notably, 27% of patients with a medial E/e′ > 15 had a mean LA pressure below 15 mmHg, indicating a significant discrepancy. In another study using the same patient population, the LA volume index (LAVI) also showed a modest correlation with mean left atrial pressure [24]. A normal LAVI with a cutoff value of 28 mL/m^2^ indicated a normal LA pressure below 15%, but had a poor specificity for elevated LA pressure > 15 mmHg. Therefore, a comprehensive approach is required for assessment of LVDD and estimating LV filling pressure, incorporating average E/e’ ratio (> 14), LAVI (> 34 mL/m^2^), pulmonary vein atrial reversal velocity (Ar-A duration ≥ 30 ms), and tricuspid regurgitation peak velocity (> 2.8 m/sec) [25].

### LA size

As LA size increases with the progression of LVDD, LA enlargement can serve as a surrogate marker for disease severity in HCM. TTE provides measurements of LA dimension and LAVI, both of which are independently associated with clinical outcomes [26, 27]. Because LA remodeling can occur asymmetrically [28], LAVI may more sensitively reflect structural changes and better predict future adverse events such as new-onset atrial fibrillation [29].

### LA strain

LA strain has emerged as a significant prognostic marker in patients with HCM [30, 31]. Impaired LA strain reflects structural remodeling and functional deterioration of the LA, in association with LVDD [32, 33]. An observational study reported that LA reservoir strain, measured by two-dimensional speckle tracking echocardiography, enhances risk stratification for future heart failure events and provide incremental prognostic value beyond the conventional LVDD grading system in patients with HCM [34]. Despite its promising prognostic utility, intervendor variability limits the establishment of a universally accepted cutoff value [35]. Furthermore, it should be noted that the LA strain-based approach has not yet been validated against invasive hemodynamic measurements. Currently, CMR studies have also demonstrated the usefulness of CMR-derived LA strain for additional risk stratification in HCM [36, 37].

### Stress echocardiography

Exercise echocardiography (diastolic stress test) may be useful for unmasking symptoms and detecting abnormal increases in LV filling pressure in patients with HCM. Exercise can be performed using either a treadmill or a bicycle, with TTE employed to assess diastolic function at each stage. Not only exercise-induced changes but also resting diastolic parameters, such as elevated E/e’, increased LAVI, and reduced LA reservoir strain, have been shown to correlate with exercise intolerance [38–40]. These findings suggest that impaired diastolic function at rest may reflect limited cardiac reserve during exertion.

### LV outflow tract obstruction

Dynamic LVOTO is one of the pathognomonic features of HCM, with reported prevalence ranging from 15 to 70%, depending on the population studies—typically lower in Asian cohorts [41–43]. Because LVOTO is associated with an increased risk of heart failure and mortality, its assessment is a critical component of HCM evaluation [44]. TTE plays a central role in identifying and monitoring LVOTO throughout the course of disease management.

**Fig. 6:**
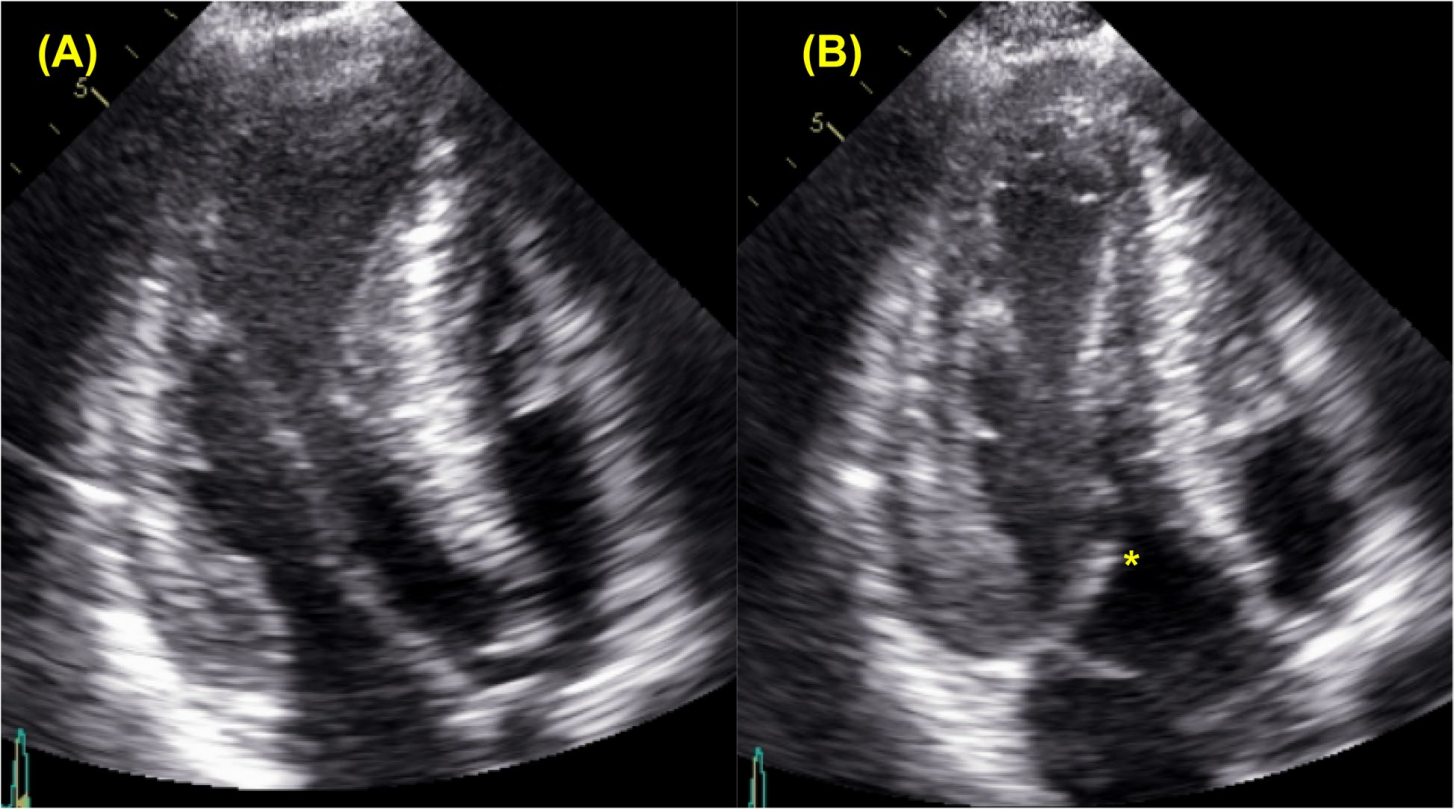
Transthoracic echocardiography of patients with obstructive hypertrophic cardiomyopathy. Apical three-chamber view at (**A**) end-diastole and **B** during systole demonstrate systolic anterior motion of the mitral valve (asterisk)

**Fig. 7:**
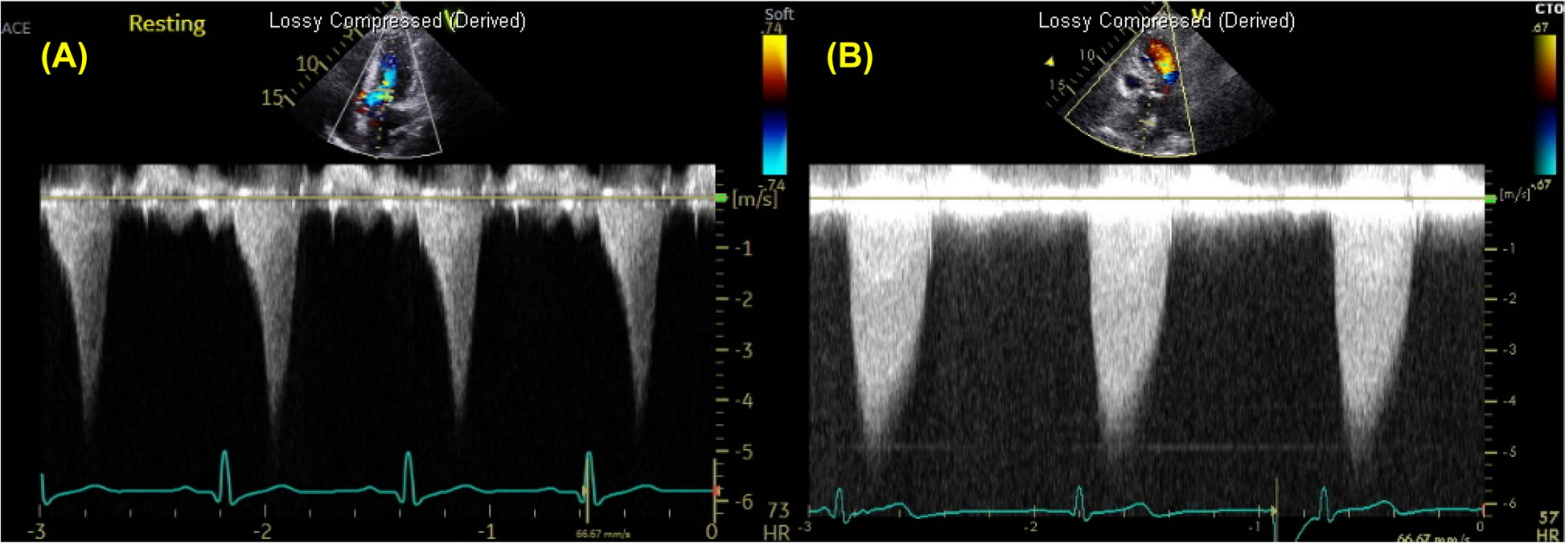
Continuous-wave Doppler demonstrates (**A**) the characteristic “dagger-shaped” profile in a patient with hypertrophic cardiomyopathy and dynamic left outflow tract obstruction, compared to (**B**) the relatively rounded profile seen in aortic stenosis

**Fig. 8:**
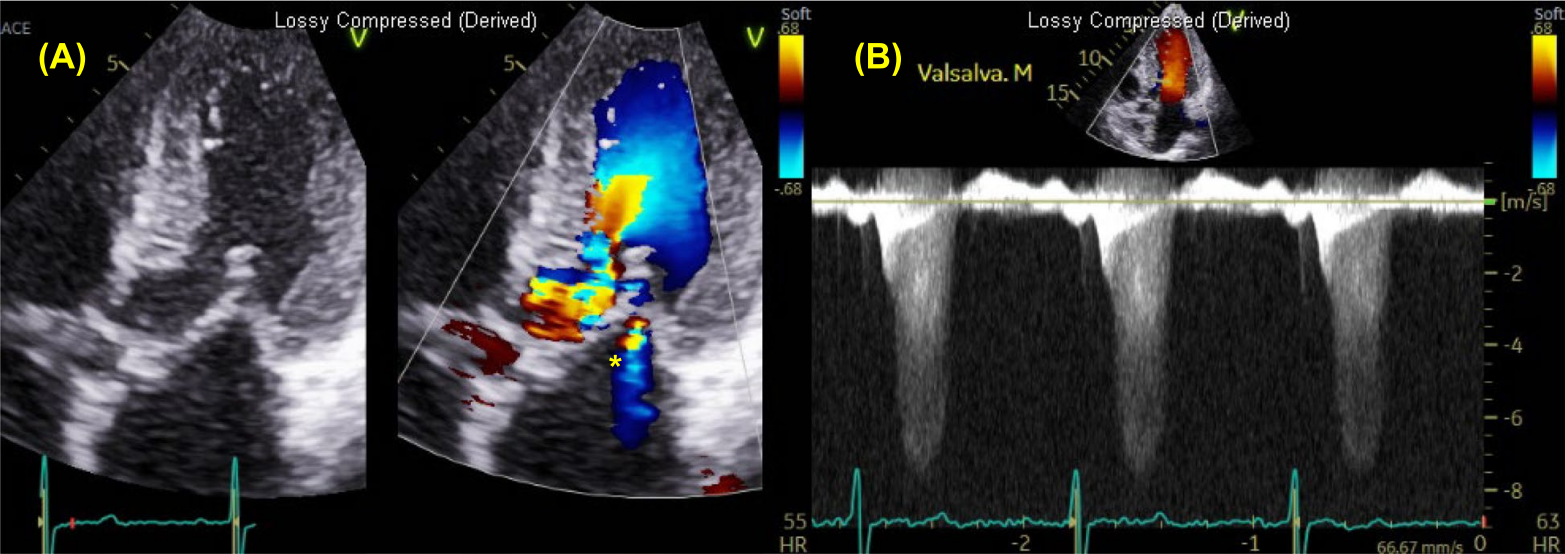
A patient with obstructive hypertrophic cardiomyopathy demonstrates (**A**) both mitral regurgitation as well as dynamic left ventricular outflow tract obstruction. **B** The continuous-wave Doppler signal is partially contaminated by the mitral regurgitation jet (asterisk)

**Fig. 9:**
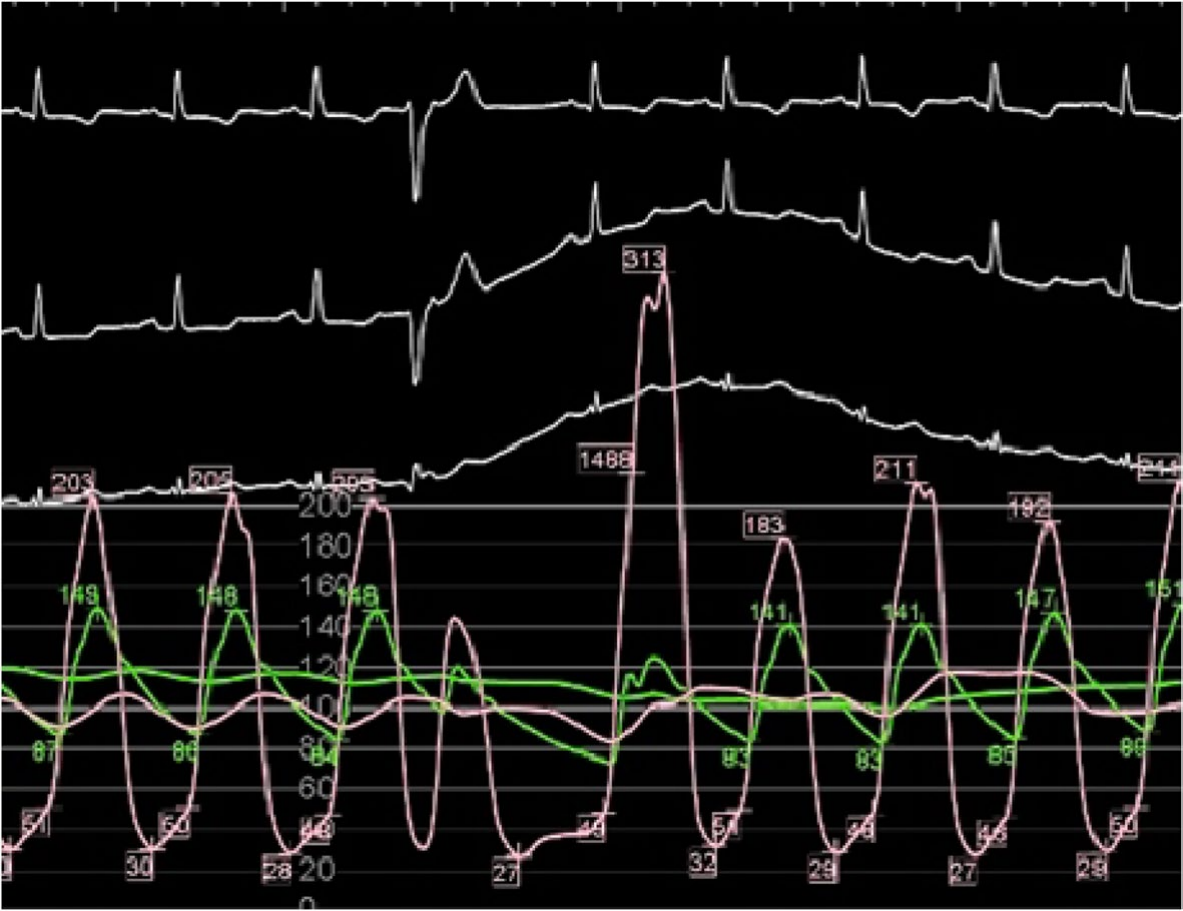
The Brockenbrough-Braunwald-Morrow sign. Simultaneous pressure tracings of the left ventricle (pink line) and aorta (green line) demonstrate a resting peak pressure gradient of approximately 50 mmHg between the left ventricle and aorta. Following a premature ventricular contraction, the pressure gradient increased markedly to 190 mmHg, accompanied by a drop in aortic pressure

**Fig. 10:**
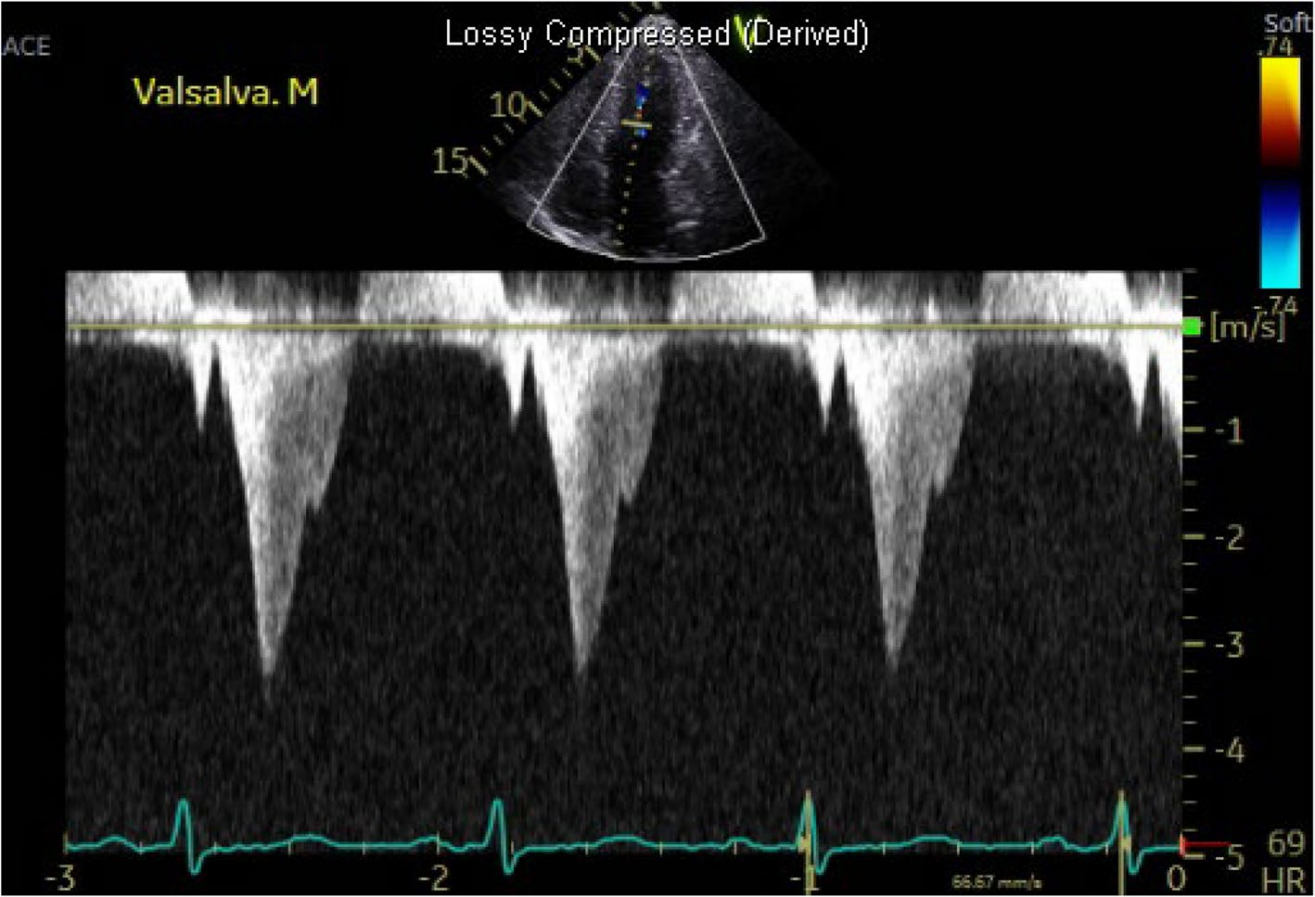
Continuous-wave Doppler demonstrates a relatively early-peaking velocity profile

Obstructive physiology is defined as instantaneous peak LV outflow tract pressure gradient ≥ 30 mmHg during systole, with ≥ 50 mmHg generally considered the threshold for therapeutic intervention. Septal hypertrophy and systolic anterior motion (SAM) of anterior mitral leaflet are the primary mechanisms driving LVOTO, largely due to the Venturi effect. SAM (with or without septal contact) can be visualized with TTE using parasternal long-axis, parasternal short-axis, or apical three-chamber views (Fig. 6, Video 2). Continuous-wave Doppler reveals a characteristic late-peaking “dagger-shaped” profile (Fig. 7). Because mitral regurgitation frequently accompanies SAM, it is important to differentiate between systolic LVOT Doppler and magnetic resonance jet to ensure accurate quantification of LVOTO (Fig. 8). Latent dynamic LVOTO, defined as the absence of significant obstruction at rest with a provoked peak LVOT gradient ≥ 30 mmHg, can be observed in a subset of patients. To reveal such obstruction, it is recommended to perform provocative maneuvers during TTE, such as the Valsalva maneuver or exercise, to unmask LVOTO and assess the maximal pressure gradient [2, 42]. In patients whom LVOTO is suspected but not clearly documented, invasive hemodynamic testing may be performed. A premature ventricular contraction can provoke a transient risk in LV systolic pressure while a simultaneous decrease in aortic pressure occurs, an observation known as the Brockenbrough-Braunwald-Morrow sign, which confirms the presence of dynamic LVOTO (Fig. 9). Mid-ventricular obliteration should be carefully differentiated from LVOTO using continuous-wave Doppler profiles and color Doppler imaging (Fig. 10, Video 3).

Multiple factors can contribute to the development of LVOTO, including elongated mitral valve leaflets, abnormal papillary muscle morphology, and anomalous basal chordal attachments [45–48]. In addition to identifying SAM, echocardiographic interpretation should carefully evaluate and report structural abnormalities potentially that may predispose to or exacerbate LVOTO.

## Tissue characterizations

While TTE describes hemodynamic information, CMR provides myocardial tissue characteristics in several ways in patients with HCM.

### Late gadolinium enhancement

**Fig. 11:**
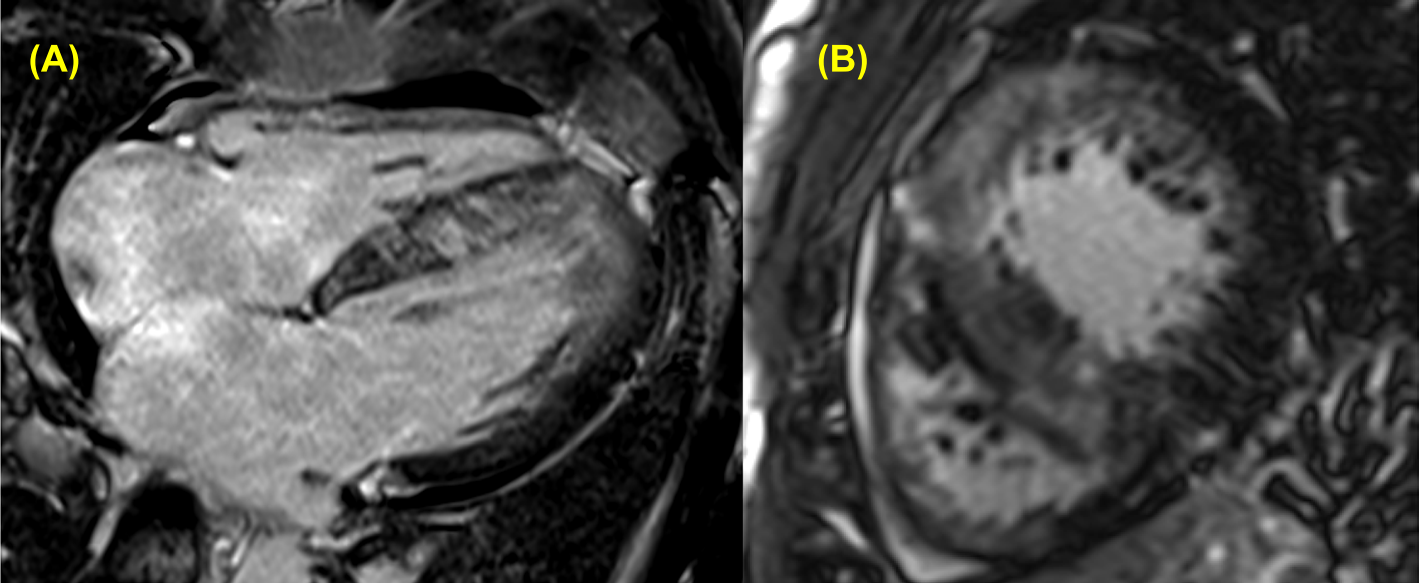
Late gadolinium enhancement in (**A**) the four-chamber view and **B** the short-axis view in a patient with hypertrophic cardiomyopathy

Fibrotic replacement in the LV myocardium is independently associated with an increased risk of sudden cardiac death in HCM [49]. CMR imaging allows for quantification of myocardial fibrosis by using late gadolinium enhancement, which is observed in over 50% of patients with HCM [50–53]. Extensive late gadolinium enhancement involving ≥ 15% of LV mass is considered a significant risk factor for sudden cardiac death and can be assessed either by quantitative gray-scale threshold techniques or by visual estimation (Fig. 11) [2, 51, 54].

### Myocardial perfusion

In patients with HCM, rest or adenosine-stress perfusion defects on CMR imaging are observed in approximately 30% to 40% of cases. These perfusion abnormalities may be related to the extent of myocardial scarring or hypertrophy and have been identified as a risk factor for an increased incidence of nonsustained ventricular tachycardia on Holter monitoring [55, 56].

### Extracellular volume

Extracellular volume reflects the extent of myocardial fibrosis within interstitial space and extracellular matrix, serving as a marker of tissue remodeling. Extracellular volume quantification requires measurement of serum hematocrit and myocardial and T1 mapping of the myocardium and blood pool following contrast administration. This technique can aid in the differential diagnosis of hypertrophied myocardium and holds potential for risk stratification in patients with HCM [57–60].

## Conclusions

Although HCM is characterized by thickened LV myocardium, the heterogeneity of its phenotype poses challenges in clinical management. A multimodal imaging approach provides more accurate diagnosis and facilitates better prognostic stratification in patients with HCM.

## Supplementary Information

Supplementary Material 1. Video 1. (A) Apical four-chamber and (B) two-chamber view of patients with hypertrophic cardiomyopathy. Video 2. Systolic anterior motion of mitral valve is observed in (A) the parasternal long-axis view and (B) the parasternal short-axis view. Video 3. Transthoracic echocardiography in a patient with mid-ventricular wall dominant hypertrophy. (A) The color Doppler image demonstrates dynamic obstruction caused by a thickened interventricular septum and hypertrophied papillary muscle. (B) Systolic anterior motion of the mitral valve is not observed in the parasternal long-axis view

## Data Availability

No datasets were generated or analysed during the current study.

## Abbreviations

CMR: Cardiac magnetic resonance
CT: Computed tomography
HCM: Hypertrophic cardiomyopathy
LA: Left atrial
LAVI: Left atrial volume index
LV: Left ventricular
LVDD: Left ventricular diastolic dysfunction
LVEF: Left ventricular ejection fraction
LVOTO: Left ventricular outflow tract obstruction
SAM: Systolic anterior motion
TTE: Transthoracic echocardiography
