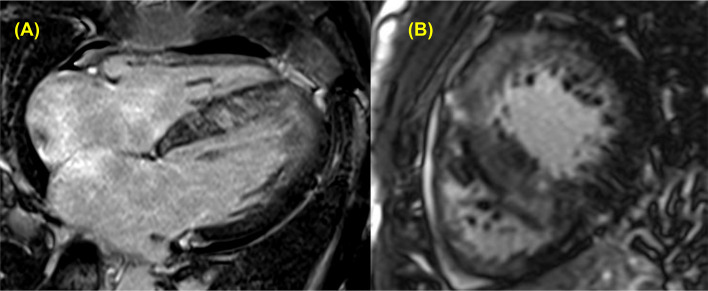# Correction: Multimodality imaging in hypertrophic cardiomyopathy

**DOI:** 10.1186/s44348-026-00067-8

**Published:** 2026-05-28

**Authors:** Jihoon Kim, Sang-Chol Lee

**Affiliations:** Division of Cardiology, Department of Internal Medicine, Heart Stroke Vascular Institute, Samsung Medical Center, Sungkyunkwan University School of Medicine, Seoul, Republic of Korea

**Correction: J Cardiovasc Imaging 34, 1 (2026)**

**https://doi.org/10.1186/s44348-025-00060-7**

After publication of this article [1], it was brought to our attention that Figure 6 and Figure 7 are incorrect and should be replaced. The asterisk in Figure 11 should be removed. The figures are shown below. The original paper has been updated.

The incorrect figure 6:
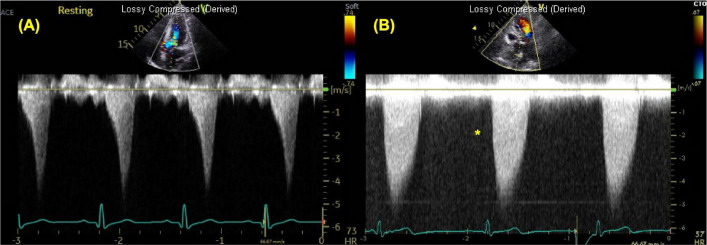


The correct figure 6:
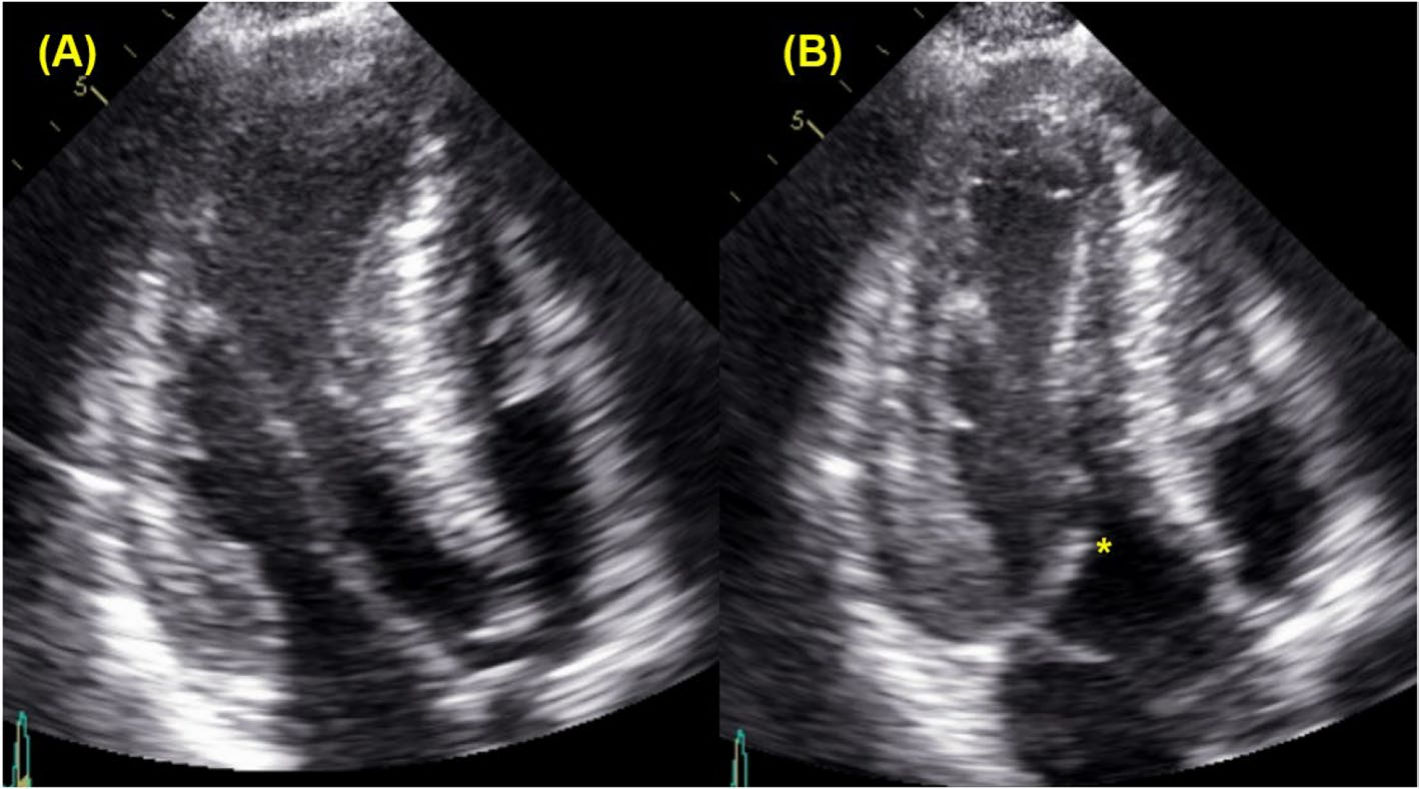


The incorrect figure 7:
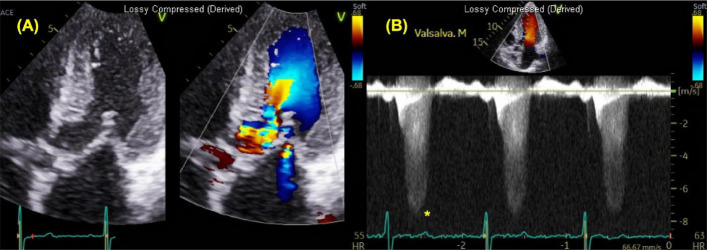


The correct figure 7:
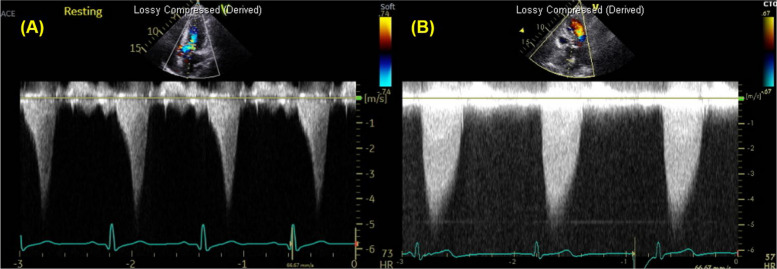


The incorrect figure 11:
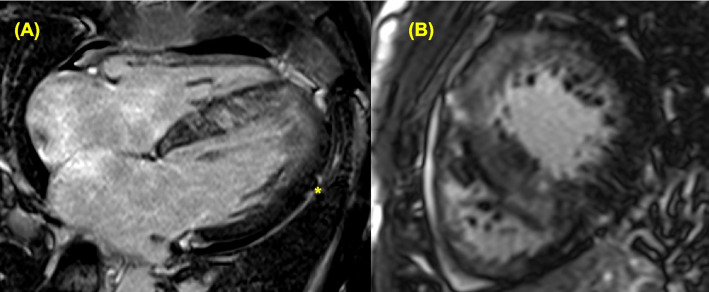


The correct figure 11: