# Supplementary figures and images for: Multimodality imaging in hypertrophic cardiomyopathy
Source: J Cardiovasc Imaging. 2026 Jan 7;34:1. doi: 10.1186/s44348-025-00060-7 (PMC12776975; doi:10.1186/s44348-025-00060-7)

## Slide 1
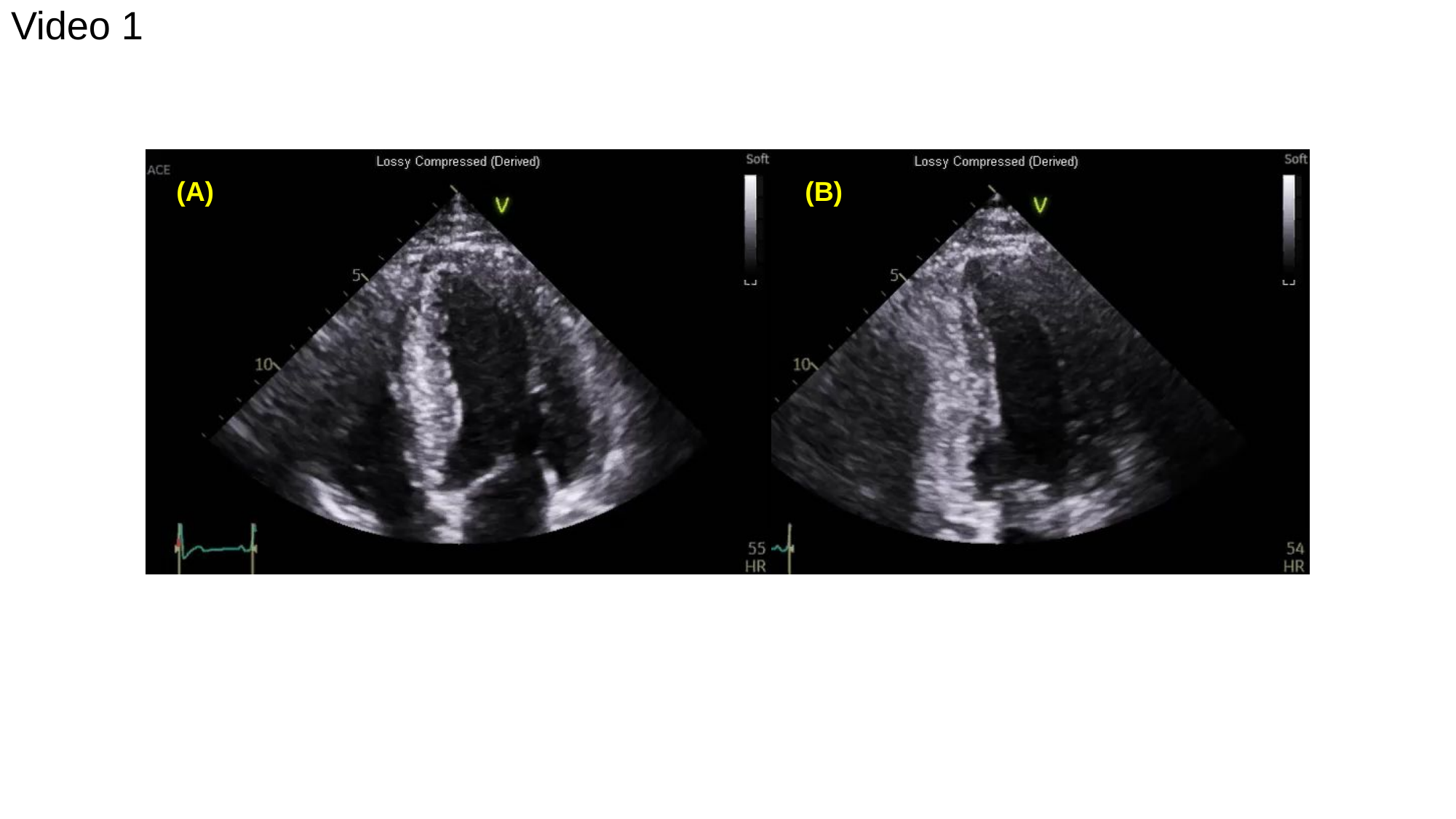

# Video 1
(A)
(B)

## Slide 2
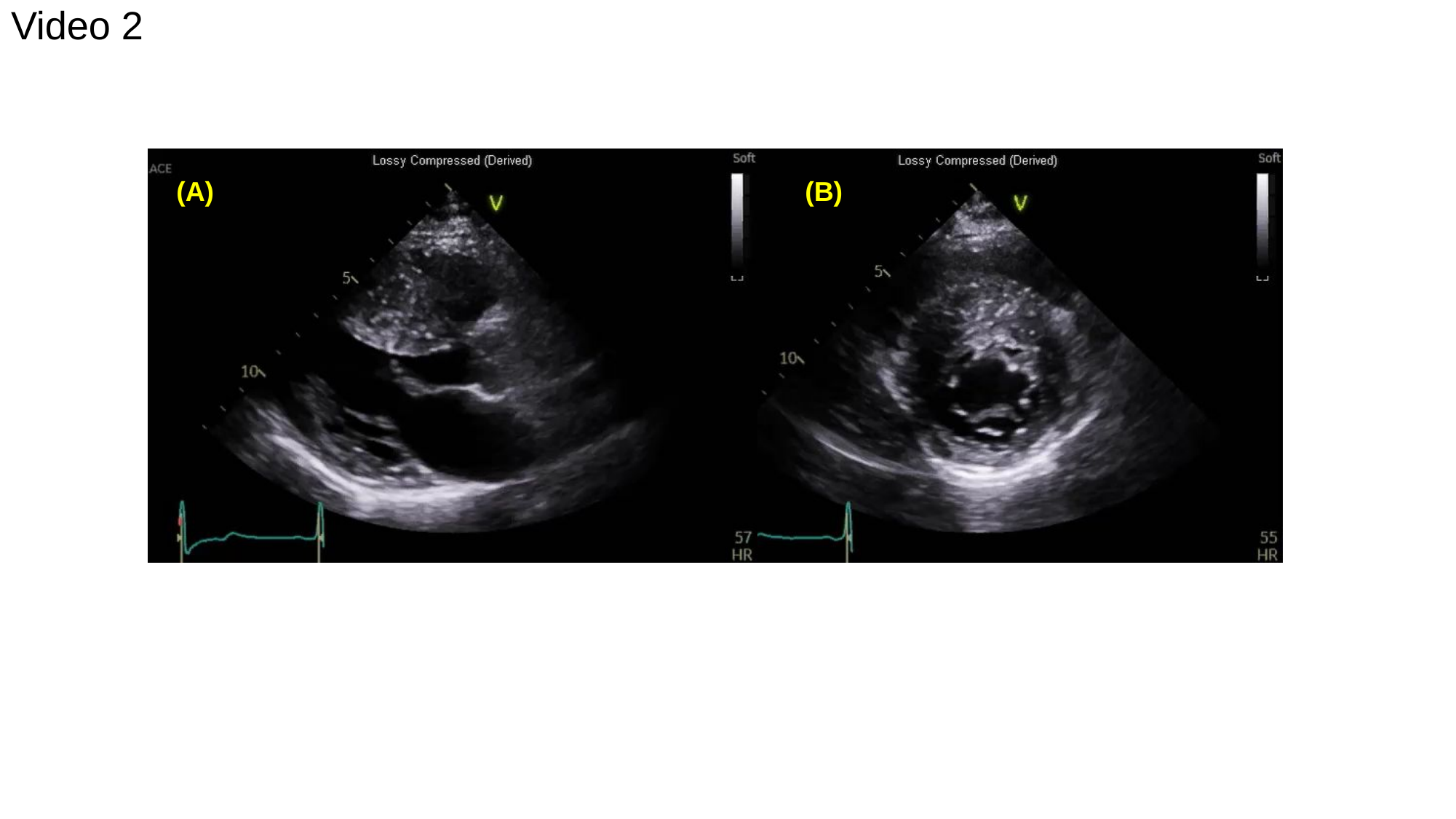

# Video 2
(A)
(B)

## Slide 3
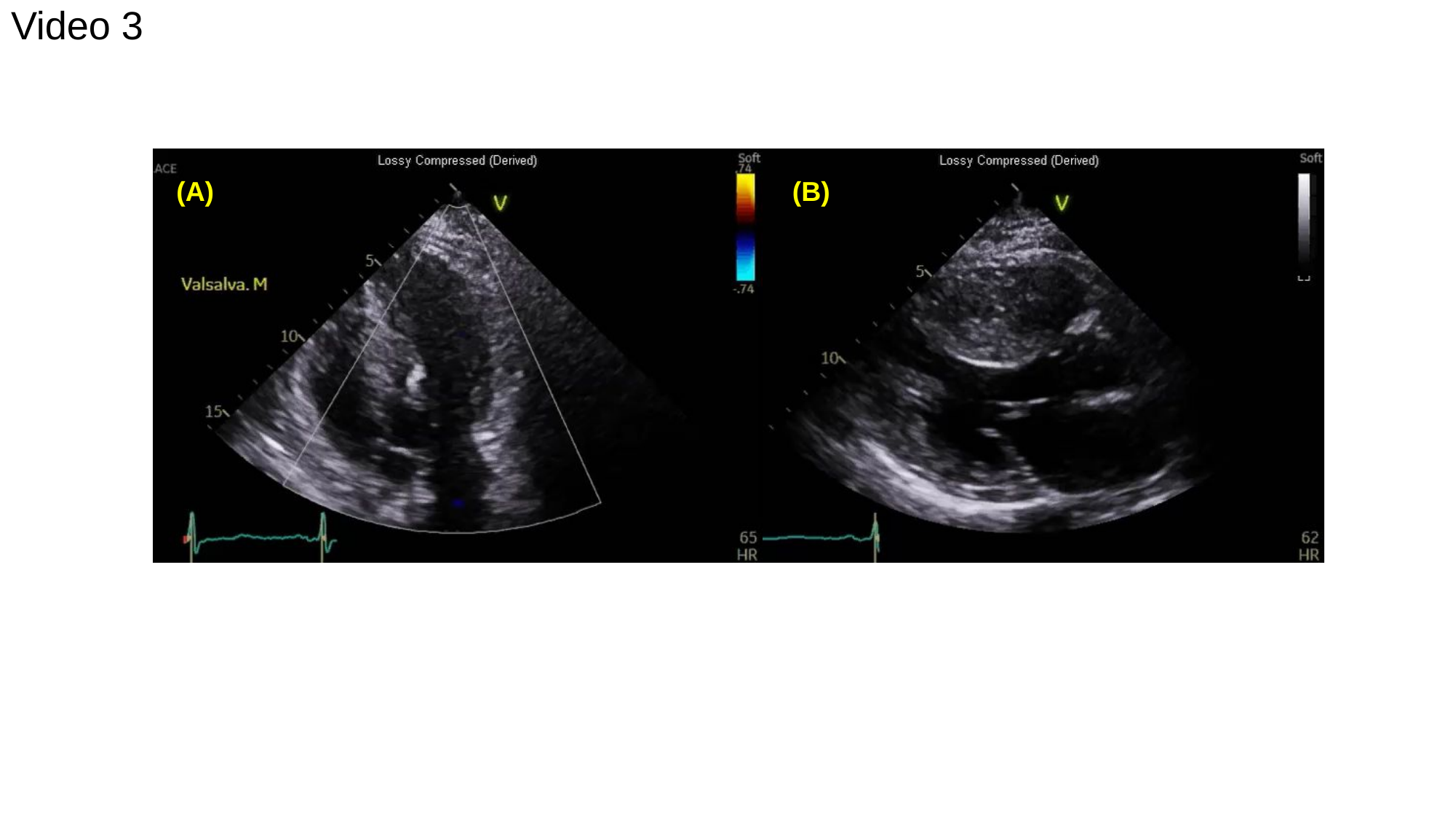

# Video 3
(A)
(B)

Supplement: Supplementary file 1 — Supplementary Material 1. Video 1. (A) Apical four-chamber and (B) two-chamber view of patients with hypertrophic cardiomyopathy. Video 2. Systolic anterior motion of mitral valve is observed in (A) the parasternal long-axis view and (B) the parasternal short-axis view. Video 3. Transthoracic echocardiography in a patient with mid-ventricular wall dominant hypertrophy. (A) The color Doppler image demonstrates dynamic obstruction caused by a thickened interventricular septum and hypertrophied papillary muscle. (B) Systolic anterior motion of the mitral valve is not observed in the parasternal long-axis view [file 44348_2025_60_MOESM1_ESM.pptx]
